# Rod Bipolar Cells Require Horizontal Cells for Invagination Into the Terminals of Rod Photoreceptors

**DOI:** 10.3389/fncel.2019.00423

**Published:** 2019-09-18

**Authors:** Lena Nemitz, Karin Dedek, Ulrike Janssen-Bienhold

**Affiliations:** ^1^Visual Neuroscience, Department of Neuroscience, University of Oldenburg, Oldenburg, Germany; ^2^Animal Navigation/Neurosensorics, Institute for Biology and Environmental Sciences, University of Oldenburg, Oldenburg, Germany; ^3^Research Center Neurosensory Science, University of Oldenburg, Oldenburg, Germany

**Keywords:** vision, retina, synapse formation, synaptogenesis, ribbon synapse, photoreceptors, horizontal cells, bipolar cells

## Abstract

In the central nervous system, neuronal processing relies on the precisely orchestrated formation of synapses during development. The first synapse of the visual system is a triad synapse, comprising photoreceptors, horizontal cells and bipolar cells. During the second postnatal week, the axon terminal processes of horizontal cells invaginate rod spherules, followed by rod bipolar cell dendrites. Both elements finally oppose the synaptic ribbon (the release site of glutamate). However, it has not been fully elucidated whether horizontal cells are essential for rod bipolar cell dendrites to find their way into the rod terminal. In the present study, we investigated this question by specifically ablating horizontal cells from the early postnatal mouse retina. We monitored the formation of the rod-to-rod bipolar cell synapse during retinal maturation until postnatal day 21. Based on quantitative electron microscopy, we found that without horizontal cells, the dendrites of rod bipolar cells never entered rod terminals. Furthermore, rods displayed significantly fewer and shorter presynaptic ribbons, suggesting that glutamate release is decreased, which coincided with significantly reduced expression of postsynaptic proteins (mGluR6, GPR179) in rod bipolar cells. Collectively, our findings uncover that horizontal cells are indeed necessary guideposts for rod bipolar cells. Whether horizontal cells release diffusible guidance cues or provide structural guidance by expressing specific cell adhesion molecules remains to be seen.

## Introduction

The establishment of functional neuronal circuits is crucial for information processing in the central nervous system and requires the formation of precise synaptic connections between different neurons. However, the mechanisms by which specific synapses are assembled are not fully understood.

At the first synapse of the visual system, photoreceptors transfer light information to horizontal cells and bipolar cells. While horizontal cell processes and ON bipolar cell dendrites invaginate into the photoreceptor terminals and form so-called triad synapses, OFF bipolar cells make non-invaginating contacts at the base of the photoreceptor terminal (Haverkamp et al., 2000). In contrast to rod spherules, which are connected to the axon terminals of horizontal cells, cone pedicles are contacted by horizontal cell dendrites (Peichl and González-Soriano, 1994). The temporal sequence of triad formation is well described in the mouse retina (Blanks et al., 1974; Rich et al., 1997; Sherry et al., 2003). Rod synaptogenesis is initiated at postnatal day 8 (P8) by the invasion of rod terminals into the outer plexiform layer (OPL). The ribbon, an electron-dense structure surrounded by tethered synaptic vesicles, is attached to the rod membrane and the terminal is contacted by a single horizontal process. Beginning at P9, a second horizontal cell process is recruited and both processes invaginate into the terminal, assuming the positions lateral to the presynaptic ribbon. One day later, one or two ON bipolar cell dendrites start to enter the terminal as the central elements of the triad. Cones synaptogenesis follows the same sequence of events but is initiated 3–4 days earlier. Triad formation is complete for rods and cones by eye opening around P14.

Several studies using knock-out mice have identified molecules that play important roles in the assembly of photoreceptor ribbon synapses. The cell adhesion protein ELFN1, which is expressed in rods, interacts transsynaptically with mGluR6, the glutamate receptor subunit of ON bipolar cells, and is essential for the selective wiring of rods and rod bipolar cells (Cao et al., 2015). Furthermore, rod photoreceptors express SynCAM1, which has also been shown to promote the invagination of ON bipolar cell dendrites (Ribic et al., 2014). Another crucial element for synapse formation between photoreceptors and ON bipolar cells is the transsynaptic interaction between the dystroglycan-pikachurin complex of photoreceptors and the orphan receptor GPR179 of ON bipolar cells (Orlandi et al., 2018). Horizontal cells have been reported to express the cell adhesion molecule NGL-2 (Soto et al., 2013, 2018) and the transmembrane semaphorin Sema6A and its receptor PlexinA4 (Matsuoka et al., 2012), which promote horizontal cell invagination into the rod terminal. Moreover, horizontal cells have been shown to help maintaining the triad synapse (Sonntag et al., 2012; Keeley et al., 2013; Wu et al., 2013; Chaya et al., 2017). Two previous studies have attempted to elucidate the role of horizontal cells in the formation of the OPL in the mouse retina. Keeley et al. (2013) revealed that horizontal cells are not essential for the initial targeting of photoreceptor terminals and bipolar cell dendrites to the OPL by using *Lim1* conditional knock-out mice, in which horizontal cells are partially mispositioned to the inner retina. In addition, Wu et al. (2013) reported that photoreceptor terminals of *Oc1* knock-out mice, that lack ∼80% of horizontal cells, contained less invaginations and displayed a loss of the classic triadic organization of postsynaptic processes. These defects were already present at P16, shortly after triad formation is completed in wild-type mice, suggesting that horizontal cells might play an important role in the assembly of photoreceptor ribbon synapses. However, it still remains unclear to which extent synaptic contacts between photoreceptors and ON bipolar cells are formed in the absence of horizontal cells, as the removal of horizontal cells from the OPL has never been complete and synapse assembly has never been studied during development.

In the present study, we investigated the role of horizontal cells in the assembly of the rod-to-rod bipolar cell synapse by specifically ablating horizontal cells from the early postnatal mouse retina via diphtheria toxin receptor (DTR)-mediated cell knock-out. We monitored the formation of the rod synapse in the absence of horizontal cells using quantitative electron microscopy and immunohistochemistry. Our analysis revealed that invaginating (rod) ON bipolar cell dendrites were completely absent from horizontal cell-deficient rod terminals. Furthermore, synaptic ribbon assembly was disrupted and the expression of the postsynaptic proteins mGluR6 and GPR179 at the dendritic tips of rod bipolar cells was strongly reduced. These findings demonstrate that horizontal cells are critical for synapse formation between rods and rod bipolar cells.

## Materials and Methods

### Animals

The generation of Cx57-DTRfrtCre mice has been described previously (Sonntag et al., 2012). Cx57-DTRfrtCre mice can be obtained from the European Mouse Mutant Archive. Animals were housed on a 12 h light/dark cycle with water and food ad libitum. For the experiments, mice of either sex were used. All procedures were performed in accordance with the law on animal protection (*Tierschutzgesetz*) issued by the German Federal Government and approved by the local animal welfare committee (*Niedersächsisches Landesamt für Verbraucherschutz und Lebensmittelsicherheit*).

### DT Injections

Control (*Cx57*^+/+^) and *Cx57*^+/DTR^ mice were injected intraperitoneally with 12.5 to 20 ng diphtheria toxin (DT, Sigma) at P4 and P5.

### Tissue Preparation

Mice were killed by decapitation (P8, P11) or deeply anesthetized with CO_2_ and killed by cervical dislocation (P15, P21). DT treatment did not affect eye opening around P14 of *Cx57*^+/+^ and *Cx57*^+/DTR^ mice. Eyes were enucleated and cornea, lens and vitreous body were removed in physiological phosphate buffered saline (pH 7.4). For immunohistochemistry, dissected eyecups were fixed in 2% paraformaldehyde (PFA) and 3% sucrose in 0.1 M phosphate buffer (PB, pH 7.4) for 20 min at room temperature (RT). For electron microscopy, retinae were isolated from the eyecups and fixed in 1% PFA, 3% sucrose and 2.5% glutaraldehyde in 0.05 M PB overnight at 4°C, washed in 0.1 M PB (3 × 30 min) and post-fixed in 1% OsO_4_ in 0.1 M PB for 1 h at RT.

### Immunohistochemistry and Image Acquisition

After fixation, eyecups of *Cx57*^+/+^ (*n* = 3–6 for each developmental stage) and *Cx57*^+/DTR^ (*n* = 3–6 for each developmental stage) mice were washed in 0.1 M PB (3 × 10 min) and cryoprotected with 30% sucrose in 0.1 M PB overnight at 4°C. The following day, tissue was embedded in Tissue-Tek O.C.T. Compound (Sakura Finetek) and sectioned vertically at 20 μm using a Leica CM1860 cryostat. Cryosections were blocked with 5% ChemiBLOCKER (Millipore), 0.3% Triton X-100 and 0.02% NaN_3_ in 0.1 M PB for 1 h at RT and incubated with primary antibodies in blocking solution overnight at 4°C. A list of primary antibodies is given in Table 1. After washing in 0.1 M PB (3 × 10 min), sections were incubated with secondary antibodies in blocking solution for 2 h at RT, washed again in 0.1 M PB (3 × 10 min) and mounted in Vectashield (Vector Laboratories). Secondary antibodies used were from donkey or goat and conjugated to either Alexa 488 or Alexa 568 (1:600, Thermo Fisher Scientific).

**TABLE 1:** List of primary antibodies used in this study.

**Antibody**	**Host, type**	**Dilution**	**Source, Catalog #, RRID**
Calbindin D-28k	Rabbit, polyclonal	1:500	Swant, CB-38, AB_2721225
Cone arrestin	Rabbit, polyclonal	1:1,000	Millipore, AB15282, AB_1163387
CtBP2	Mouse, monoclonal	1:5,000	BD Biosciences, 612044, AB_399431
Dihydropyridine-sensitive calcium channel α1 subunit	Mouse, monoclonal	1:500	Millipore, MAB427, AB_2069582
mGluR6	Sheep, polyclonal	1:100	Gift from Kirill A. Martemyanov (Cao et al., 2011)
PKCα	Goat, polyclonal	1:500	R&D Systems, AF5340, AB_2168552
PKCα	Mouse, monoclonal	1:500	Santa Cruz Biotechnology, sc-80, AB_628141
PSD-95	Mouse, monoclonal	1:5,000	NeuroMab, 75-028, AB_2307331

Images were acquired using a Leica TCS SP8 confocal laser scanning microscope. Scanning was performed with an HC PL APO CS2 63×/1.4 oil objective at a resolution of 1024 × 1024 pixels and a *z*-axis increment of 0.2 μm. Maximum projections of collapsed confocal stacks are shown. Brightness and contrast were adjusted using Fiji (Schindelin et al., 2012).

### Electron Microscopy

Fixed retinae were washed in 0.1 M PB (3 × 10 min), dehydrated in a series of 50 to 100% acetone and incubated in a 1:1 mixture of acetone and Agar 100 Resin (Agar Scientific) for 1 h at RT. After incubation in pure Agar 100 Resin overnight at RT, embedding medium was hardened for 48 h at 60°C. Embedded retinae were cut using a Reichert-Jung Ultracut E ultramicrotome. Semithin sections (0.5 μm thickness) were collected on slides, stained with 2% toluidine blue and 0.5% sodium borate in double-distilled water and examined with a Leica DM6 microscope. Ultrathin sections (90 nm thickness) were collected on copper grids and analyzed using a Zeiss EM 902A electron microscope. Electron micrographs were adjusted in brightness and contrast using Adobe Photoshop CS6 Extended (Adobe Systems).

### Quantification and Statistical Analysis

To quantify the OPL thickness in toluidine blue-stained semithin sections of *Cx57*^+/+^ (*n* = 3 for P8, *n* = 4 for P11, *n* = 5 for P15, *n* = 3 for P21) and *Cx57*^+/DTR^ (*n* = 3 for P8, *n* = 4 for P11, *n* = 3 for P15, *n* = 2 for P21) retinae, we measured the distance from outer nuclear layer (ONL) to inner nuclear layer (INL) somata at 10 locations per animal using the line tool in Fiji. For comparison of rod synaptogenesis in *Cx57*^+/+^ (*n* = 4 for P11, *n* = 3 for P15) and *Cx57*^+/DTR^ mice (*n* = 4 for P11, *n* = 5 for P15), we analyzed between 746 and 1177 rod terminal profiles for each genotype and developmental stage and classified them into four categories: (1) empty terminals (profiles without invaginations), (2) monads (profiles with one invaginating horizontal cell process), (3) dyads (profiles with two invaginating horizontal cell processes) and (4) triads (profiles with at least one invaginating ON bipolar cell dendrite). Rod terminal profiles that could not be clearly assigned to one of these groups were categorized as unclassified. To compare the frequency of rod terminals with and without ribbons between genotypes, the same set of rod terminal profiles was analyzed. For the quantification of ribbon sizes in wild-type (*n* = 3) and horizontal cell-ablated (*n* = 5) retinae at P15, we measured the length of 30 ribbons per animal using the line tool in Fiji. To compare mGluR6- and GPR179-positive puncta between *Cx57*^+/+^ (*n* = 3) and *Cx57*^+/DTR^ (*n* = 5) mice at P15, confocal stacks were deconvolved with a theoretical point spread function using Huygens Essential deconvolution software (SVI) and further processed in Fiji. Background-subtracted single scans were used for the analysis. Intensity thresholds were set manually and kept constant for both experimental groups. Number and average size of puncta were determined for 9 regions of interest (46.08 × 25.25 μm, covering the OPL and proximal ONL) per animal using the *Analyze particles* function in Fiji. Particles <0.81 and >22.50 μm^2^ were excluded from the analysis to prevent the inclusion of noise and stained blood vessels.

Statistical analysis was performed using GraphPad Prism 5 (GraphPad Software). A *p* value <0.05 was considered statistically significant. Data are presented as mean ± standard deviation (SD).

To compare OPL thickness, ribbon length and the number and average size of mGluR6- and GPR179-positive puncta between *Cx57*^+/+^ and *Cx57*^+/DTR^ mice, Wilcoxon rank sum test was used. Differences in rod synaptogenesis stages and frequencies of ribbons in rod terminals were analyzed using χ*^2^* test and Fisher’s exact test, respectively.

## Results

### Early Postnatal Horizontal Cell Ablation Causes Defects in the Development of the OPL

**FIGURE 1:**
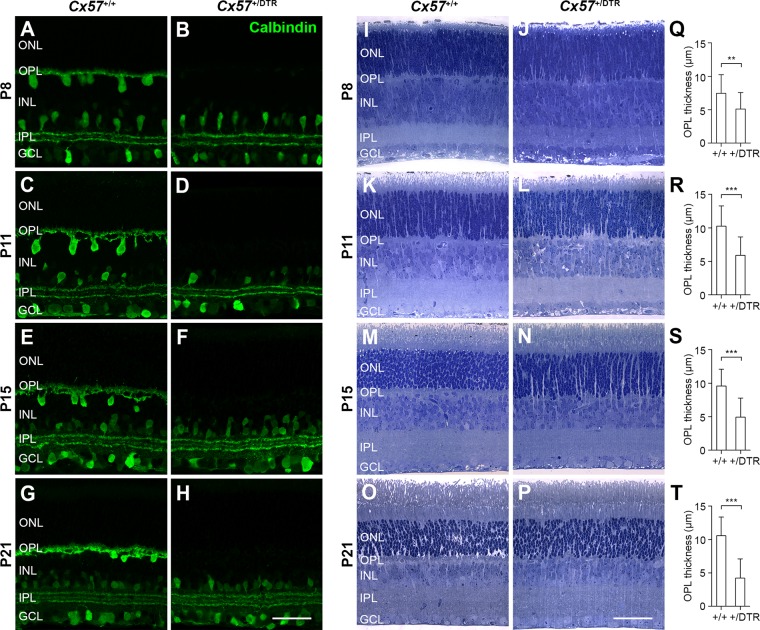
Eliminating horizontal cells during early postnatal development results in defective formation of the OPL. **(A–H)** Retinal cryosections of DT-injected *Cx57*^+/+^ and *Cx57*^+/DTR^ mice were stained for calbindin, a horizontal cell marker. In *Cx57*^+/DTR^ retinae, calbindin labeling of horizontal cells was strongly reduced at P8 **(B)** and completely missing as early as P11 **(D,F,H)**. The gross morphology of calbindin-immunoreactive amacrine and ganglion cells was unaltered. **(I–P)** Toluidine blue staining of retinal semithin sections from *Cx57*^+/+^ and *Cx57*^+/DTR^ mice. The OPL of horizontal cell-ablated retinae was markedly thinner from P8 to P21 compared to wild-type retinae. The thickness of all other retinal layers was similar for both genotypes. **(Q–T)** Quantification of OPL thickness in *Cx57*^+/+^ (*n* = 3 for P8, *n* = 4 for P11, *n* = 5 for P15, *n* = 3 for P21) and *Cx57*^+/DTR^ (*n* = 3 for P8, *n* = 4 for P11, *n* = 3 for P15, *n* = 2 for P21) mice. *p* < 0.01 (P8), *p* < 0.0001 (P11, P15, P21), Wilcoxon rank sum test. Values are presented as mean ± SD. ^∗∗^*p* < 0.01, ^∗∗∗^*p* < 0.001. Scale bars, 50 μm.

Previous attempts to ablate horizontal cells during retinal development by intravitreal injection of kainic acid at the day of birth (Messersmith and Redburn, 1990) and oncogene expression under the control of the phenylethanolamine *N*-methyltransferase promoter (Hammang et al., 1993; Peachey et al., 1997) were not cell class-specific and the elimination of horizontal cells from the OPL by knock-out of *Lim1* (Poche et al., 2007; Keeley et al., 2013) and *Oc1* (Wu et al., 2013) did not affect the whole horizontal cell population. In the rabbit retina, the ablation of horizontal cells alters the segregation of retinal cells into distinct laminae and increases the rod-to-cone ratio (Messersmith and Redburn, 1990). To minimize any potential impact of horizontal cell ablation on retinal lamination and cell differentiation, we here ablated horizontal cells after they had migrated to their final position in the distal part of the inner nuclear layer but immediately before their axon terminal processes invaginate into the rod terminal. For the ablation, we used Cx57-DTRfrtCre mice (Sonntag et al., 2012) that express the primate DTR under the control of the horizontal cell-specific connexin57 (Cx57) promoter and intraperitoneally administered diphtheria toxin (DT) at postnatal day 4 (P4) and P5. To verify the loss of horizontal cells, we labeled retinal cryosections with an antibody against the horizontal cell marker calbindin (Haverkamp and Wässle, 2000). Calbindin immunoreactivity was drastically reduced in horizontal cells at P8 (Figure 1B) and completely absent from the outer retina from P11 onward (Figures 1D,F,H), indicating that the entire cell class was successfully ablated. The gross morphology of calbindin-positive amacrine and ganglion cells was comparable to that in the control (Figures 1A–H), demonstrating that the ablation is specific and restricted to horizontal cells.

To investigate the effects of early postnatal horizontal cell ablation on the development of the overall morphology of the retina, we stained retinal semithin sections of DT-injected *Cx57*^+/+^ and *Cx57*^+/DTR^ mice with toluidine blue (Figures 1I–P). Compared with wild-type retinae, horizontal cell-ablated retinae showed a significant reduction in the thickness of the OPL between P8 and P21 (Figures 1Q–T; P8: *p* < 0.01; P11, P15, P21: *p* < 0.0001, Wilcoxon rank sum test), suggesting that the assembly of this layer, which contains the synapses between photoreceptors, horizontal cells and bipolar cells, is defective. By contrast, all other retinal layers were similar in thickness (Figures 1I–P) and labeling for Müller cells with glutamate synthetase did not show any gross differences (Supplementary Figure S1).

### Horizontal Cells Are Required for ON Bipolar Cell Invagination in Rods

To study the role of horizontal cells in the formation of rod synapses, we examined individual rod terminal profiles in the OPL of *Cx57*^+/+^ and *Cx57*^+/DTR^ mice by electron microscopy and classified them according to their developmental stage into (1) empty terminals (profiles without invaginations), (2) monads (profiles with one invaginating horizontal cell process), (3) dyads (profiles with two invaginating horizontal cell processes) and (4) triads (profiles with at least one invaginating ON bipolar cell dendrite). For the analysis, two different time points were chosen: P11 (one day after the beginning of triad formation) and P15 (one day after triad formation is complete).

**FIGURE 2:**
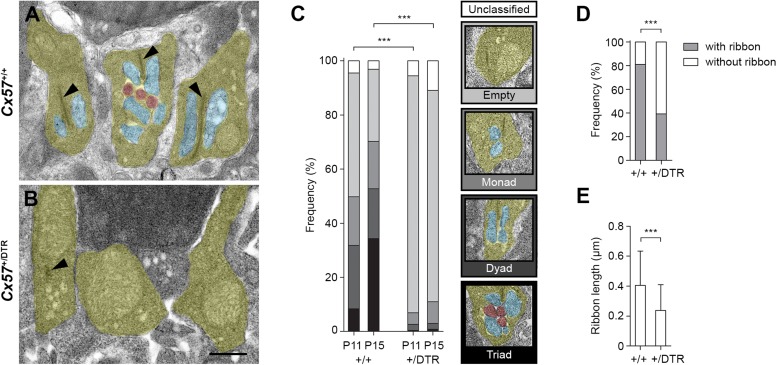
Rod terminals of horizontal cell-ablated mice lack ON bipolar cell invaginations. **(A,B)** Representative electron micrographs of rod terminals from *Cx57*^+/+^ and *Cx57*^+/DTR^ mice at P15. Rod terminal profiles (yellow) in wild-type retinae showed large presynaptic ribbons (arrowheads) and invaginations from horizontal cells (blue) and ON bipolar cells (red) **(A)**. In contrast, rod terminal profiles in horizontal cell-ablated retinae typically lacked any invaginations. Synaptic ribbons were absent or shorter (arrowhead) **(B)**. **(C)** Quantification of rod synaptogenesis stages in *Cx57*^+/+^ and *Cx57*^+/DTR^ mice at P11 and P15. Between 746 and 1177 rod terminal profiles from wild-type (*n* = 4 for P11 and *n* = 3 for P15) and *Cx57*^+/DTR^ mice (*n* = 4 for P11, *n* = 5 for P15) were analyzed for each age. Rod terminal profiles without invaginations were designated as empty, rod terminal profiles with one or two horizontal cell process were designated as monads or dyads, respectively, and rod terminal profiles containing at least one invaginating ON bipolar cell dendrite were designated as triads. *p* < 0.0001, χ*^2^* test. **(D)** Frequencies of rod terminal profiles with and without ribbons in wild-type (*n* = 3) and horizontal cell-ablated (*n* = 5) retinae. *p* < 0.0001, Fisher’s exact test. **(E)** Quantification of ribbon lengths in *Cx57*^+/+^ *(n* = 3) and *Cx57*^+/DTR^ (*n* = 5) mice. *p* < 0.0001, Wilcoxon rank rum test. Values are presented as mean ± SD. ^∗∗∗^*p* < 0.001. Scale bar, 500 μm.

The electron microscopic examination revealed that at P11, the majority of rod terminal profiles from *Cx57*^+/+^ mice were empty. A substantial portion had invaginations from either one or two horizontal cell processes and only very few terminals already displayed the classic triad configuration at this time point (Figure 2C; 46% empty, 18% monads, 23% dyads, 8% triads, 5% unclassified, analysis of 849 rod terminal profiles from *n* = 4 mice). By P15, most of the rod terminal profiles in controls contained triads and the percentage of empty terminals was decreased compared to P11, as expected (Figures 2A,C; 27% empty, 18% monads, 18% dyads, 34% triads, 3% unclassified, analysis of 746 rod terminal profiles from *n* = 3 mice). In contrast, in *Cx57*^+/DTR^ mice, the vast majority of rod terminal profiles did not show any invaginations at either developmental stage. Occasionally, we observed profiles containing remnants from horizontal cells. However, invaginating ON bipolar cell dendrites were found at a very low frequency and only in terminals with horizontal cell leftovers (Figures 2B,C; P11: 88% empty, 4% monads, 2% dyads, <1% triads, 6% unclassified, analysis of 824 rod terminal profiles from *n* = 4 mice; P15: 78% empty, 8% monads, 2% dyads, <1% triads, 11% unclassified, analysis of 1177 rod terminal profiles from *n* = 5 mice). The distribution of rod synaptogenesis stages was significantly different between *Cx57*^+/+^ and *Cx57*^+/DTR^ mice (P11, P15: *p* < 0.0001, χ*^2^* test). As we never found a rod terminal profile containing only ON bipolar dendrites (but no remnants of horizontal cells) at any age, our data strongly indicates that horizontal cells are indispensable for rod bipolar cells to invaginate into rod terminals.

In addition to the lack of invaginating contacts, rod terminal profiles of horizontal cell-ablated mice displayed less presynaptic ribbons at P15 (Figures 2A,B,D; *Cx57*^+/+^: 81% with ribbons, 19% without ribbons, analysis of 746 rod terminal profiles from *n* = 3 mice; *Cx57*^+/DTR^: 39% with ribbons, 61% without ribbons, analysis of 1177 rod terminal profiles from *n* = 5 mice; *p* < 0.0001, Fisher’s exact test). If present, ribbons in *Cx57*^+/DTR^ retinae were significantly shorter compared to ribbons in wild-type retinae (Figures 2A,B,E; *Cx57*^+/+^: 0.40 ± 0.23 μm, *n* = 3; *Cx57*^+/DTR^: 0.24 ± 0.17 μm, *n* = 5; *p* < 0.0001, Wilcoxon rank sum test).

### Horizontal Cell-Ablated Mice Display a Retraction of Rod Terminals and Disrupted Ribbon Assembly

**FIGURE 3:**
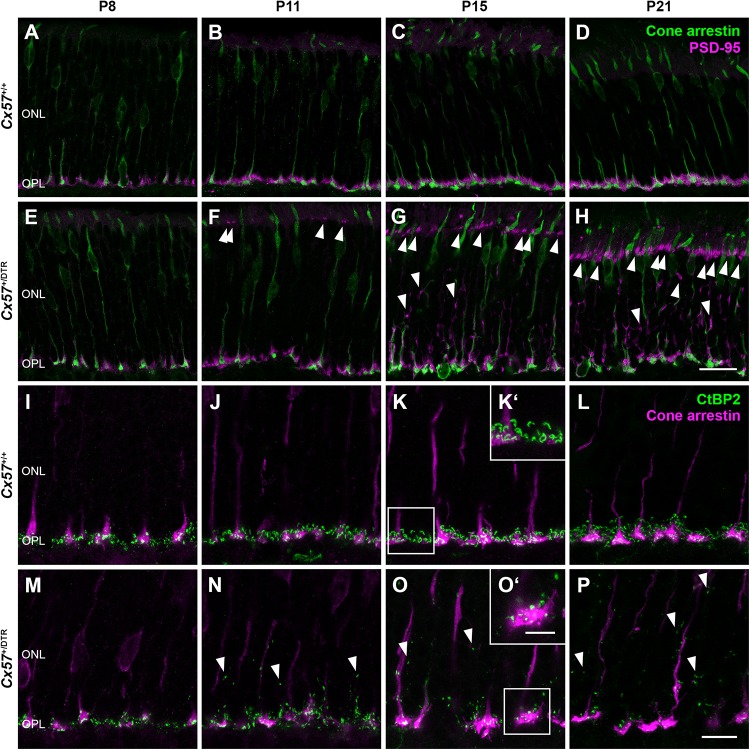
Retraction of rod terminals and disrupted ribbon assembly following early postnatal horizontal cell ablation. **(A–H)** Double labeling of retinal sections from *Cx57*^+/+^ and *Cx57*^+/DTR^ mice for cone arrestin (green), a marker for cones, and PSD-95 (magenta), a marker for photoreceptor terminals. While PSD-95-positive photoreceptor terminals from *Cx57*^+/+^ mice were confined to the OPL **(A–D)**, *Cx57*^+/DTR^ mice showed a progressive retraction of rod terminals into the ONL beginning at P11 (arrowheads) **(F–H)**. Cones terminals retained their stratification in the OPL. **(I–P)** Immunostaining of *Cx57*^+/+^ and *Cx57*^+/DTR^ retinae with antibodies against the synaptic ribbon marker CtBP2 (green) and cone arrestin (magenta). **(K‘,O‘)** Magnified images of ribbons in panels **(K,O)**. Ribbons of horizontal cell-ablated mice were mainly restricted to the OPL and horseshoe shaped from P15 onward **(I–L)**. By contrast, horizontal cell-ablated mice showed ectopic ribbons in the ONL as early as P11 (arrowheads) **(N–P)** and CtBP2 labeling displayed a punctate pattern **(M–P)**. Scale bars, 20 μm **(H)**, 10 μm **(P)** and 5 μm **(O‘)**.

The low OPL thickness in *Cx57*^+/DTR^ mice (Figures 1J,L,N,P) points to a reduced number of photoreceptor terminals within this layer. To examine their localization in the retina, we stained photoreceptor terminals and cone photoreceptors in *Cx57*^+/+^ (Figures 3A–D) and *Cx57*^+/DTR^ retinae (Figures 3E–H) with antibodies specific for PSD-95 and cone arrestin, respectively, which allowed us to discriminate between rod and cone terminals. At P8, rod spherules were already present in the OPL of *Cx57*^+/DTR^ mice (Figure 3E), growing in number until P11 (Figure 3F). This finding is in line with previous studies reporting that horizontal cells are not necessary for targeting of rod terminals to the OPL (Messersmith and Redburn, 1990; Keeley et al., 2013). In addition, we observed an increase in ectopic rod spherules in the ONL between P11 and P21 (Figures 3F–H, arrowheads), demonstrating that rods retract their axon terminals following early postnatal horizontal cell ablation. By contrast, cone terminals generally remained in the OPL (Figures 3E–H).

During photoreceptor synaptogenesis, ribbons are assembled from non-membranous precursor spheres (Regus-Leidig et al., 2009). Our ultrastructural analysis showed that synaptic ribbons were either absent from rod terminals in horizontal cell-ablated mice or shorter when compared to controls (Figures 2B,D,E, arrowhead). To confirm this finding, we labeled retinal sections from *Cx57*^+/+^ and *Cx57*^+/DTR^ mice for CtBP2, a ribbon marker, and cone arrestin, which enabled us to distinguish between ribbons of rods and cones. In wild-type retinae, CtBP2 immunolabeling was almost completely confined to the OPL (Figures 3I–L), whereas in horizontal cell-ablated retinae, we frequently found ectopic CtBP2-positive structures in the ONL (Figures 3M–P, arrowheads), in agreement with the retraction of rod terminals we observed. The typical horseshoe shape of the ribbons was clearly visible as early as P15 in the wild-type retina (Figures 3K,K‘,L). Consistent with the electron microscopic observations, CtBP2 labeling in horizontal cell-ablated mice was rather punctate, even at P15 and P21 (Figures 3O,O‘,P), suggesting that synaptic ribbon assembly is impaired after ablation of horizontal cells. This is in line with earlier reports showing that ribbons are missing or shorter in photoreceptor terminals of horizontal cell-deficient mice (Sonntag et al., 2012; Keeley et al., 2013; Wu et al., 2013).

### Recruitment of mGluR6 and GPR179 to the Dendritic Tips of Rod Bipolar Cells Is Impaired in Horizontal Cell-Ablated Mice

**FIGURE 4:**
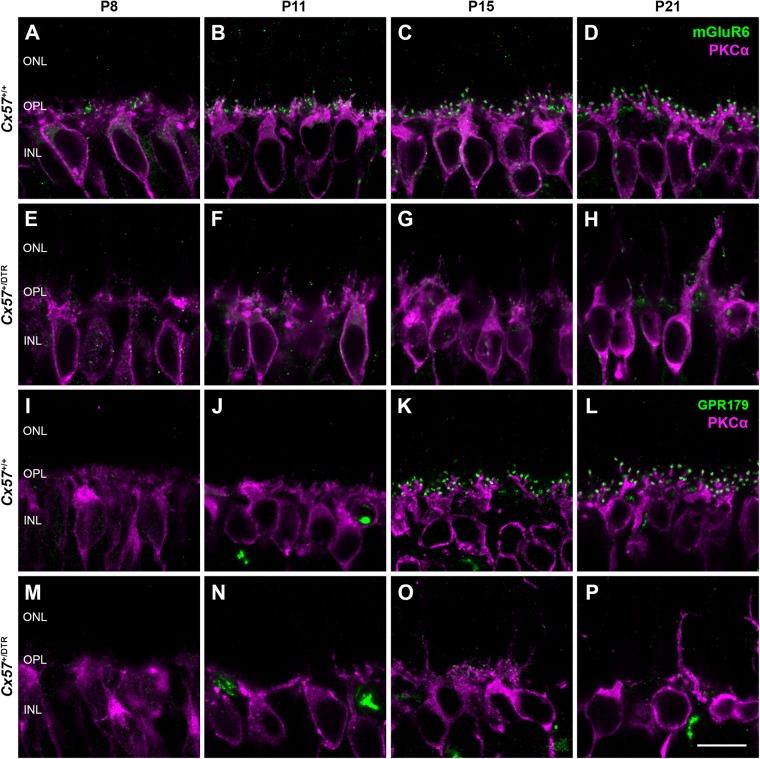
Horizontal cell ablation affects mGluR6 and GPR179 localization in rod bipolar cells. **(A–H)** Vertical sections from *Cx57*^+/+^ and *Cx57*^+/DTR^ retinae were stained for mGluR6 (green) and PKCα (magenta), a rod bipolar cell marker. In control mice, rod bipolar cell processes terminated in the OPL. Expression of mGluR6 at the dendritic tips of rod bipolar cells was already detectable at P8 and increased up to P21 **(A–D)**. By contrast, rod bipolar cell dendrites from horizontal cell-ablated mice showed an outgrowth into the ONL and a reduced mGluR6 accumulation **(E–H)**. **(I–P)** Retinae of *Cx57*^+/+^ and *Cx57*^+/DTR^ mice were double labeled with an antibody against Ca_*v*_1.1 (green), that cross-reacts with GPR179, and an antibody against PKCα (magenta). GPR179-immunoreactive puncta decorated the distal tips of rod bipolar cells as early as P15 in the wild-type retina **(I–L)**. In comparison, horizontal cell-ablated retinae displayed only very few and small GPR179-positive puncta at rod bipolar cell dendritic tips **(M–P)**. Scale bars, 10 μm.

To assess changes on the postsynaptic side, we stained rod bipolar cells with an antibody against protein kinase C alpha (PKCα) and components of the ON bipolar cell signaling complex with antibodies against the metabotropic glutamate receptor mGluR6 and Ca_*v*_1.1. The latter cross-reacts with GPR179 (Hasan et al., 2016), a G protein-coupled receptor serving as a membrane anchor for regulators of G-protein signaling (Orlandi et al., 2012). During retinal development, ON bipolar cell dendrites emerge from neuroepithelial-like processes that terminate at the outer limiting membrane (Morgan et al., 2006). By P8, rod bipolar cells from wild-type mice had already established a dendritic network in the OPL and rod bipolar cell dendritic tips were decorated with some mGluR6-immunoreactive puncta (Figure 4A). The number and size of mGluR6-positive puncta increased until the retina reached maturity at P21 (Figures 4A–D). GPR179 immunoreactivity was first detectable in the developing OPL at P15 (Figures 4I–L). Compared to wild-type mice, rod bipolar cell dendrites from *Cx57*^+/DTR^ mice were less branched and showed progressive sprouting into the ONL. Furthermore, the expression of mGluR6 and GPR179 in rod bipolar cell processes was greatly reduced throughout development (Figures 4E–H,M–P). We quantified this for P15 and found the number and average size of mGluR6- and GPR170-positive puncta significantly reduced in horizontal cell-ablated mice (Table 2). Thus, our data suggest that postsynaptic targeting of mGluR6 cascade-related elements to the dendritic tips of rod bipolar cells is compromised in the absence of horizontal cells, potentially because glutamatergic input from photoreceptors is impaired.

**TABLE 2:** Quantification of mGluR6- and GPR179-positive puncta in *Cx57*^+/+^ and *Cx57*^+/DTR^ mice at P15.

	***Cx57*^+/+^ (mean ± SD)**	***Cx57*^+/DTR^ (mean ± SD)**	***p* value**
Number of mGluR6-postive puncta per 100 μm	93.7 ± 25.4	26.14 ± 16.0	<0.0001
Average size of mGluR6-positive puncta (in μm^2^)	0.12 ± 0.03	0.08 ± 0.02	<0.0001
Number of GPR179-positive puncta per 100 μm	82.9 ± 25.6	36.5 ± 27.5	<0.0001
Average size of GPR179-positive puncta (in μm^2^)	0.12 ± 0.03	0.07 ± 0.01	<0.0001

## Discussion

**FIGURE 5:**
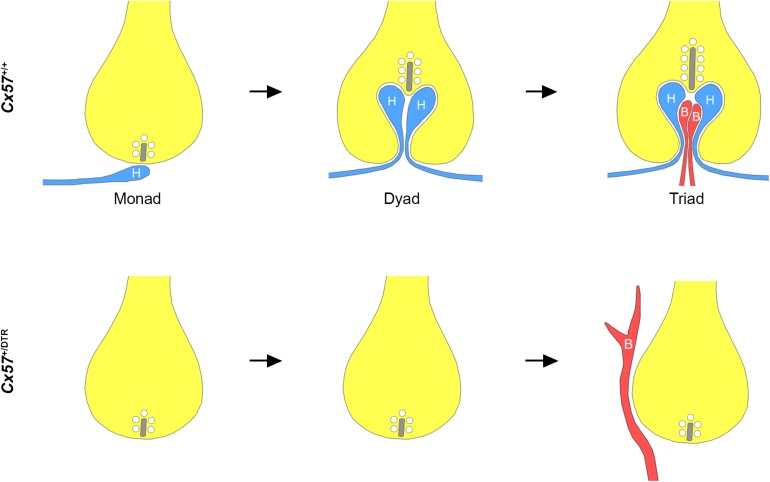
Schematic illustration of rod synaptogenesis in wild-type and horizontal cell-ablated mice. In the wild-type retina, the rod terminal is initially contacted by a single horizontal cell process (H). The following day, another horizontal cell process is recruited and both invaginate into the terminal, occupying the positions lateral to the presynaptic ribbon. Subsequently, one or two ON bipolar cell dendrites (B) insert between the two horizontal cell processes. In the absence of horizontal cells, rod bipolar cell dendrites are not able to invaginate into the rod terminal and sprout into the ONL. Synaptic ribbons, if present, are shorter compared to wild-type mice.

In this study, we investigated the role of horizontal cells in the formation of the rod photoreceptor synapse by ablating horizontal cells from the early postnatal mouse retina. Our immunohistochemical and electron microscopic analysis revealed that in the absence of horizontal cells (1) ON bipolar cells were not able to invaginate into rod terminals, (2) presynaptic ribbon assembly was disrupted and (3) postsynaptic accumulation of mGluR6 signaling complex components in ON bipolar cells was impaired. These results demonstrate that horizontal cells are indispensable for the assembly of the rod-to-rod bipolar cell synapse (Figure 5).

Keeley et al. (2013) investigated the role of horizontal cells in the formation and maintenance of the OPL using *Lim1* conditional knock-out mice, in which horizontal cells are partially mislocalized in the inner retina. Consistent with our results, this study revealed that horizontal cells are not necessary for the initial targeting of photoreceptor terminals and bipolar cell dendrites to the OPL but for the maintenance of this layer. A study by Wu et al. (2013) showed that the knock-out of the homeodomain transcription factor *Oc1* results in a loss of ∼80% of the horizontal cell population. Comparable to *Cx57*^+/DTR^ mice, *Oc1* knock-out mice displayed fewer and shorter ribbons, which were rarely recruited to the plasma membrane. Moreover, photoreceptor terminals did not show the stereotypic triad organization at P16 and P30. However, some invaginations of postsynaptic processes were present in rod and cone terminals, likely because ∼20% of horizontal cells are still formed in *Oc1* knock-out mice. In addition to that, it has been shown that also long-term removal of horizontal cells from the adult retina leads to a disruption of triad synapses and remodeling in the outer retina (Sonntag et al., 2012; Chaya et al., 2017). Thus, horizontal cells are not only essential for the formation (this study), but also for the maintenance of synaptic contacts between rods and rod bipolar cells (Sonntag et al., 2012; Keeley et al., 2013; Wu et al., 2013; Chaya et al., 2017).

But why are rod bipolar cells not able to find their way into the rod terminal in the absence of horizontal cells? We consider three mechanisms by which horizontal cells may contribute to synapse formation between rods and rod bipolar cells. First, horizontal cells may release guidance cues that direct pathfinding of rod bipolar cell dendrites. Earlier studies have shown that glial cells provide attractive and repellant cues and act as guidepost cells and intermediate targets for neurons (reviewed in Chotard and Salecker, 2004). As Müller cells, the most abundant retinal glial cells, are among the last cell types born during neurogenesis (Young, 1985; Rapaport et al., 2004), horizontal cells possibly take over this function of glial cells in the developing retina.

Second, horizontal cells may provide structural guidance for rod bipolar cell dendrites to invaginate into the rod terminal by expressing specific cell adhesion molecules. This hypothesis is supported by the finding that emerging bipolar cell dendrites in and outside the OPL are closely associated with processes from horizontal cells in the first postnatal week (Morgan et al., 2006). Moreover, it has been reported that rod bipolar cell dendrites grow along and co-fasciculate with sprouting horizontal cell processes in CNGA3/CNGB1 double knock-out (Michalakis et al., 2013) and bassoon mutant mice (Specht et al., 2009). In horizontal cells, only a few cell adhesion molecules have been identified so far. NGL-2 localizes to horizontal cell axons, directs their laminar targeting and promotes synapse formation between rods and horizontal cells (Soto et al., 2013, 2018). However, in NGL-2-deficient mice, photoreceptor terminals and bipolar cell dendrites stratify normally and photoreceptor ribbon synapses are formed, albeit at a lower frequency (Soto et al., 2013), indicating that this molecule is not responsible for the developmental defects we observed in horizontal cell-ablated mice. In addition, N-cadherin has been shown to be important for horizontal cell dendrite morphogenesis and synapse formation in the chicken retina (Tanabe et al., 2006) but it remains to be tested whether N-cadherin also plays a role in synaptogenesis the mouse retina. Apart from that, it is possible that horizontal cells influence the expression of cell adhesion or extracellular matrix proteins in rod photoreceptors, such as ELFN1 (Cao et al., 2015), SynCAM1 (Ribic et al., 2014) or the dystroglycan-pikachurin complex (Sato et al., 2008; Omori et al., 2012; Orlandi et al., 2018), that promote the invagination of rod bipolar cells.

Third, disturbed neurotransmission in the outer retina may impede synapse formation between rods and rod bipolar cells in horizontal cell-ablated mice. Horizontal cells receive glutamatergic input from photoreceptors and provide feedback signals to photoreceptors (Mangel, 1991; Wu, 1991; Thoreson et al., 2008; Jackman et al., 2011) and feedforward signals to bipolar cells (Yang and Wu, 1991; Fahey and Burkhardt, 2003). Different mechanisms have been proposed for horizontal cell feedback: hemichannel-mediated ephaptic feedback (Kamermans, 2001), pH-mediated feedback (Hirasawa and Kaneko, 2003), GABA-mediated feedback (Wu, 1992) and a combination of all three (Kemmler et al., 2014). Feedforward signaling to bipolar cells possibly involves GABA (Puller et al., 2014). Several studies have reported that neurotransmission is important for the maturation and maintenance of photoreceptor ribbon synapses. Similar to horizontal cell ablation, mutations in presynaptic proteins such as bassoon (Dick et al., 2003; Specht et al., 2009), CAST (tom Dieck et al., 2012), CaBP4 (Haeseleer et al., 2004), Ca_*v*_1.4 (Mansergh et al., 2005; Chang et al., 2006; Bayley and Morgans, 2007; Liu et al., 2013; Zabouri and Haverkamp, 2013) and CNGA3/CNGB1 (Michalakis et al., 2013) that regulate glutamate release from photoreceptors cause a loss of synaptic contacts in the OPL, a retraction of rod terminals and sprouting of horizontal and bipolar cell processes. This may be caused by a lack of synaptic transmission and/or lack of horizontal cell signals. However, when the light-dependent modulation of feedback and feedforward signals from horizontal cells is abolished, this has no effects on the structure of the rod-to-rod bipolar cell synapse (Ströh et al., 2018). Also, the elimination of GABA synthesis in horizontal cells does not prevent the assembly of photoreceptor ribbon synapses (Schubert et al., 2010), arguing against a direct involvement of horizontal cell signaling in synapse formation or maintenance.

Whether horizontal cells are not only essential for rod synapse formation, but also for cone synapse formation remains to be seen. As cone synaptogenesis is initiated 3–4 days prior to rod synaptogenesis (Rich et al., 1997; Sherry et al., 2003) (at the same time we induced the horizontal cell ablation by DT injections), our approach is not suitable to investigate the assembly of the cone-to-ON bipolar cell synapse in the absence of horizontal cells. In contrast to rod spherules, which are invaginated by the axon terminals of horizontal cells, cone pedicles are invaginated by horizontal cell dendrites (Peichl and González-Soriano, 1994). Thus, synapse assembly might be differently regulated for both photoreceptor types. However, ribbon structures and postsynaptic recruitment of mGluR6 signaling complex components seemed equally affected in the rod and cone pathway, suggesting that horizontal cells may also be critical for cone synapse formation.

Although further investigations are needed to identify the underlying mechanism, our study demonstrates that horizontal cells are essential for presynaptic photoreceptor maturation, invagination of rod bipolar cells into rods and postsynaptic recruitment of ON bipolar cell signaling components in the outer retina.

## Data Availability

All datasets generated for this study are included in the manuscript and/or the Supplementary Files.

## Ethics Statement

The animal study was reviewed and approved by Niedersächsisches Landesamt für Verbraucherschutz und Lebensmittelsicherheit.

## Author Contributions

UJ-B performed DT injections and tissue preparation. LN performed immunohistochemistry, image acquisition, electron microscopy, quantification and statistical analysis, prepared the figures, and wrote a first draft of the manuscript. All authors designed the experiments and contributed to the interpretation of data. KD and UJ-B revised the manuscript.

## Conflict of Interest Statement

The authors declare that the research was conducted in the absence of any commercial or financial relationships that could be construed as a potential conflict of interest.
