# Supplementary material for: Rod Bipolar Cells Require Horizontal Cells for Invagination Into the Terminals of Rod Photoreceptors
Source: Front Cell Neurosci. 2019 Sep 18;13:423. doi: 10.3389/fncel.2019.00423 (PMC6760018; doi:10.3389/fncel.2019.00423)
Supplement: Supplementary file 1 [file Table_1.DOCX]

Supplementary Material

# Supplementary Data

# Supplementary Figures and Tables

## Supplementary Figures


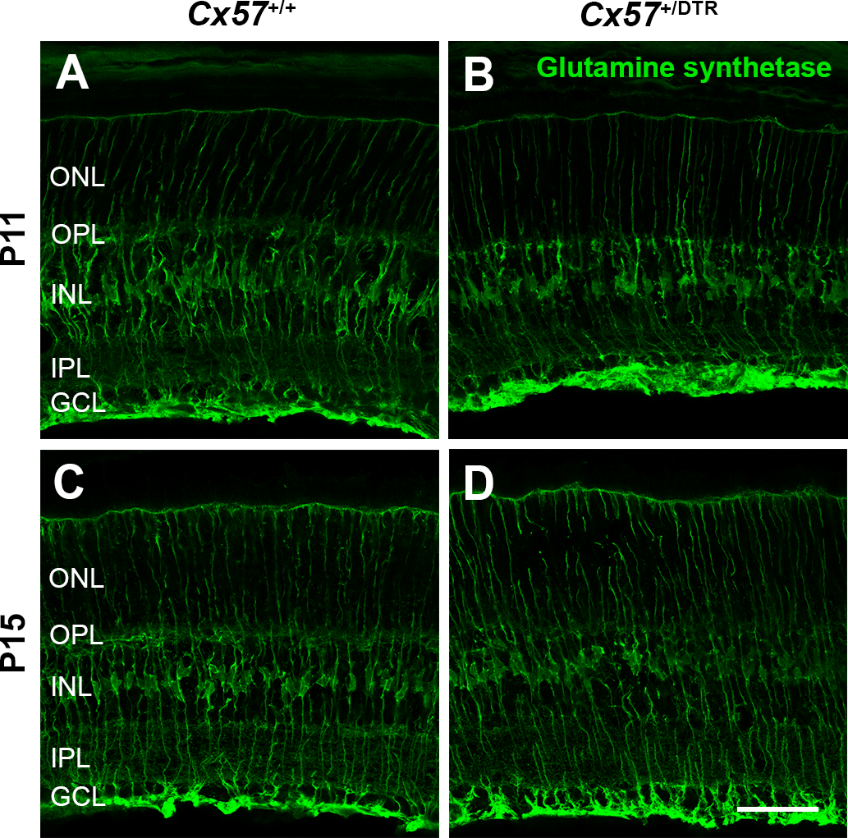


**Supplementary Figure 1.** **Early postnatal horizontal cell ablation has no effects on the gross morphology of Müller cells.** **(A-D)** Vertical cryosections of *Cx57*^+/+^ and *Cx57*^+/DTR^ retinae were stained with an antibody against glutamine synthetase (BD Biosciences, Catalog #: 610517, RRID: AB_397879, 1:2000), a Müller cell marker. The gross morphology of Müller cells in horizontal cell-ablated mice was comparable to that in control mice. Scale bar, 50 µm.
